# Pediatric Patient With Neurofibromatosis I Presenting With Perceptual Disturbances and a Suicide Attempt

**DOI:** 10.7759/cureus.62237

**Published:** 2024-06-12

**Authors:** Erin M Sanzone, Kaitlin Sanzone, Zoe Tirado, Anthony Rostain, Maju Koola

**Affiliations:** 1 Physical Medical and Rehabilitation, Cooper Medical School at Rowan University, Camden, USA; 2 Psychiatry, University of San Diego, San Diego, USA; 3 Psychiatry, Cooper University Hospital, Camden, USA; 4 Psychiatry and Behavioral Health, Cooper University Hospital, Camden, USA

**Keywords:** psychosis, perceptual disturbances, nfi, neurofibromatosis, neurofibromas

## Abstract

This is a case of a pediatric patient with a history of neurofibromatosis I (NFI) presenting to the emergency department secondary to a suicide attempt via self-strangulation after being verbally and physically bullied at school. Upon hospital admission, the 10-year-old patient was found to have significant auditory and visual perceptual hallucinations in addition to suicidal ideations, for which psychiatry was consulted. The patient underwent magnetic resonance imaging (MRI) of the brain to evaluate for intracranial neurofibromas as a potential etiology of his behavior. There is evidence that the growth of neurofibromas in the brain can be associated with psychosis. His brain MRI was significant for multiple foci of non-enhancing lesions seen in the cerebellum, white matter, supratentorial white matter, and bilateral hippocampi that can be seen in NFI, highlighting a medical etiology for the patient’s auditory and visual perceptual disturbances. The objective of this case report is to explore medical causes of psychosis including metabolic disorders, neurodegenerative diseases, metabolic disturbances, parathyroid diseases, genetic disorders (Fragile X, Prader-Willi, etc.), autoimmune disorders, multiple sclerosis, temporal lobe epilepsy, infections, and brain tumors.

## Introduction

This case report is about a patient is a 10-year-old boy with a medical history of neurofibromatosis I (NFI), attention-deficit/hyperactivity disorder (ADHD), and auditory and visual hallucinations with 20 previous suicide attempts (including self-suffocation, stabbing neck, etc.) presenting to the emergency department for a suicide attempt. NFI is an inherited neurocutaneous disorder that is associated with bone abnormalities, vasculopathy, café au lait spots (coffee-colored patches on the skin), neurofibromas (benign tumor of peripheral nerves), and cognitive impairment [1]. NFI has been found to present with more psychiatric and mood disorders than the general population, likely due to social and physical disabilities as well as alterations in cognition [1]. Brain imaging in patients with NF1 compared to controls without NF1 has shown increased gray matter volumes in the thalamus, corpus striatum, dorsal midbrain, and cerebellum bilaterally [2]. Researchers have hypothesized these brain findings correlated with low memory load, learning disabilities, and ADHD in nearly 45% of patients and autism spectrum disorder in nearly 25% of the population [3]. Stimulants have been shown to help ADHD symptoms in NFI individuals [4]. Perceptual disturbances are perceptions that can affect a person’s ability to process sensory information [5]. Perceptual disorders can range from hallucinations, delusions, disorganized thinking, and heightened sensitivity, which all prevent a person from recognizing objects, people, or sounds [4]. Perceptual disturbances are considered a positive symptom of psychosis. In this case report, the patient developed auditory hallucinations. Psychosis is defined as a syndrome characterized by hallucinations and delusions [6]. Many providers initially consider primary psychotic disorders, such as schizophrenia, when creating a differential diagnosis of psychosis. However, it is imperative to have strong clinical suspicion about secondary medical causes of psychosis before diagnosing patients with a primary psychotic disorder. Medical causes of psychosis are described as identifiable brain diseases, including metabolic disorders, neurodegenerative diseases, metabolic disturbances, parathyroid diseases, genetic disorders (Fragile X, Prader-Willi, etc.), autoimmune disorders, multiple sclerosis, temporal lobe epilepsy, infections, brain tumors, and neurofibromatosis [7]. Another differential diagnosis to consider in a patient with a traumatic history is trauma-induced psychosis. When there is a high clinical suspicion of a medical cause, such as in this case of a young pediatric patient with a predisposing genetic condition presenting with perceptual disturbances, it is prudent to perform a thorough investigation into all potential medical etiologies to properly treat the patients.

## Case presentation

The patient is a 10-year-old boy with a medical history of neurofibromatosis I (NFI), attention-deficit/hyperactivity disorder (ADHD), and previous suicide attempts who presented to the emergency department (ED) in March of 2023 with his parents after a suicide attempt by holding his breath with his palm after being bullied by a classmate. The patient made statements about suicidal ideation at school, his parents were called to pick him up, and they brought him to the ED. The patient also endorsed auditory and visual perceptual disturbances, stating that the auditory perceptions occur almost every day and the visual disturbances occur more than three times per week. He stated he has heard derogatory voices for the past few years that constantly tell him, “You are worthless, no one likes you, you should hurt yourself, you should die.” He stated that the voices are a major contributor to his suicide attempts. At the time of the examination, the patient did not appear to be responding to internal stimuli. The psychiatry consultation-liaison (CL) team was consulted to assess the etiology of the presenting symptoms and to provide management recommendations.

The patient had a history of 15-20 suicide attempts over the past five years. The first attempt was when he was seven years old; the most recent was two months earlier. Methods employed included self-suffocation, stabbing himself in the neck, placing a bag over his head, and eating silica pouches. There was no family history of suicide attempts, completed suicide, or primary psychotic disorders. At seven years old, he was evaluated by ED psychiatry services, but he had never been in outpatient therapy or treated with psychotropic medication. The patient stated that his suicide attempts always occurred at school when his peers called him upsetting names. He reported that over the past few years, kids in his old school would bully him, push him down, and beat him up. He stated, “Even my sister doesn't like me and calls me ugly.”

The patient endorsed past and current symptoms of depression, stating he felt sad most days. Per his mother, the patient could not stay still, talked during class, had increased reactivity, and had poor concentration. She also reported a history of destroying property and hitting dogs.

The patient was first suspected of having NFI at age eight months due to the presence of multiple café au lait macules on his back and torso and mild hypotonia with motor delays. He was sequentially followed until receiving an official diagnosis at 21 months, at which point he had an increased number of café au lait macules, macrocephaly, hypotonia, and a broadened gait.

At the time of hospitalization, the patient was in the fifth grade and living at home with his mother and two sisters. He reported that he felt safe at home. He reportedly did not have a good relationship with his biological father and did not live with him. The patient had significant traumatic experiences in his childhood, including physical abuse and episodes of bullying throughout his life. He had difficulty forming relationships and trusting others.

Following the initial interview, the CL team deemed it appropriate to refer the patient for transfer to a child and adolescent psychiatry unit (CAPU) for his safety. He was admitted to the general pediatrics hospitalist service while awaiting an available CAPU bed, and the psychology consult service serially reevaluated him for additional diagnostic evaluation and management recommendations. The patient had a negative urinalysis and toxicology screen to rule out infectious and substance-induced psychosis. He remained on the pediatrics floor for eight days, at which time he had stabilized enough for disposition to the ambulatory care environment. In the days following admission, the patient began exhibiting increasingly hyperactive behaviors, including flickering the lights during interviews and throwing pillows. He demonstrated increasingly concerning perceptual disturbances and was noted to be responding to internal stimuli during subsequent interviews.

On examination, the patient stated that his visual perceptual disturbances occurred about three times per week and had been ongoing for the past few years. He described these disturbances as “ghost figures” in which some are evil, and others are friendly. During the interview, the patient was noted to interact verbally and behaviorally with “ghost people.” He explained that ghost people were largely friends and protectors, while shadow people told him bad things about himself and others and tried to harm him and control his body. He described them as the “Devil and Satan” who said bad things about him and tried to control his body. He believed the shadow people were controlling his body while he was in the principal's office on the day of his hospitalization. They were moving his hand toward the shredder, an action that was only aborted when a teacher entered the room and intervened.

At this point in his admission, the CL team was increasingly concerned about the auditory and visual perceptual changes the patient was describing and whether they were related to a primary psychotic disorder or psychosis due to a general medical condition.

**Figure 1:**
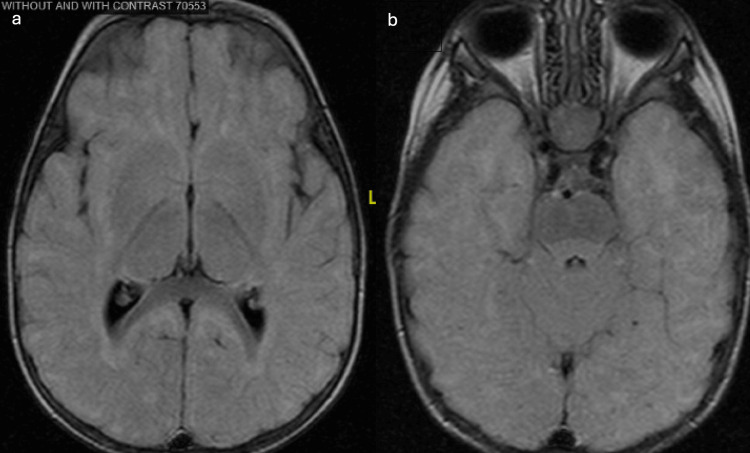
Brain magnetic resonance imaging in 2013 a. MRI results demonstrate a grossly normal examination of the brain. There is no evidence of restricted diffusion, abnormal susceptibility, or suspicious enhancement. No focus of abnormal parenchymal signal is demonstrated, indicating a normal degree of myelination for the patient’s age. b. Incidentally noted is mucosal thickening of the right greater than left maxillary sinuses. There is mild hypertrophy of the adenoids and enlargement of the palatine tonsils.

**Figure 2:**
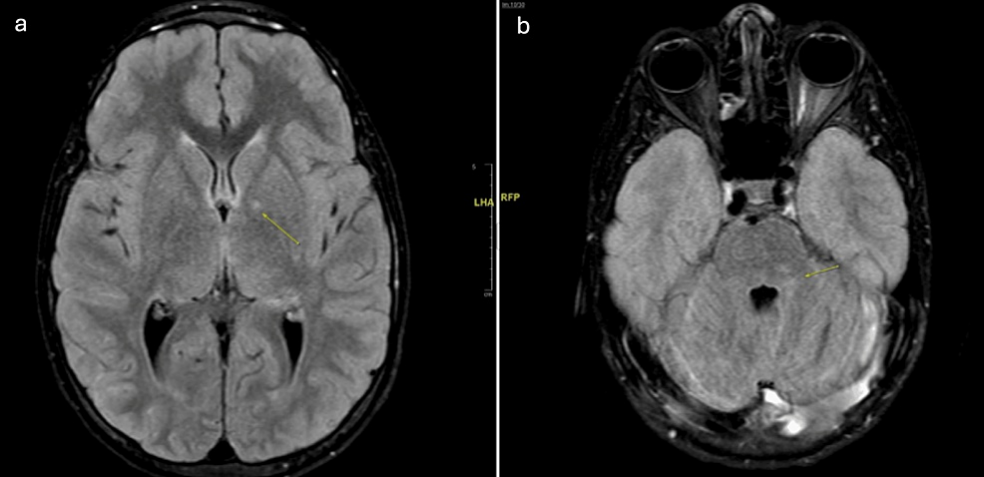
Brain magnetic resonance imaging with neurofibromatosis I in 2023 a. MRI results were significant for multiple T2/FLAIR hyperintense foci seen in the cerebellum, supratentorial white matter, and bilateral hippocampi, which are findings commonly associated with NFI.13. Of note, no enhancing lesions were seen on post-contrast sequences. As seen in Figure 1a, the MRI FLAIR sequences demonstrate increased signal in the bilateral hippocampi and a small focus within the left globus pallidus. b. There is a small focus of T2 and FLAIR hyperintense signal within the left cerebellar white matter. The neurofibromas themselves are not seen within the brain on imaging, as they are peripheral nerve sheath tumors, but there can be focal areas of signal intensity that are bright on T2/FLAIR; however, these do not enhance on post-contrast sequences. FLAIR: fluid-attenuated inversion recovery

Given the patient’s young age and the absence of a family history of psychosis, primary psychosis was considered to be less likely [6]. It was also theorized that given the patient’s history of traumatic childhood events of physical abuse in foster care, he could be having dissociative-like features and perceptual disturbances secondary to post-traumatic stress disorder [8]. There is increasing evidence suggesting childhood trauma can manifest as psychosis later in life [9]. Population-based studies have found that childhood trauma can lead to negative perceptions of self, negative affect, and psychotic symptoms, as well as dysregulation of cortisol and increased sensitivity to stressors [9]. Given the patient’s lifelong history of NFI, the CL team also considered the possibility that neurofibroma growth was contributing to the psychiatric behavioral manifestations. Current research suggests that NFI is often associated with psychiatric disorders, which are more frequent in patients with NFI (in one-third of patients) than in the general population [1]. Therefore, magnetic resonance imaging (MRI) of the brain was recommended to rule out psychosis secondary to a medical condition before treating a psychiatric disorder. Of note, the patient’s prior MRI was performed in 2013 (at 8 months of age), as seen in Figure 1. The findings from the patient’s current brain MRI are shown in Figure 2.

At this point, the CL team aimed to address the patient’s three major psychiatric concerns: (1) perceptual disturbances, (2) hyperactivity/impulsivity, and (3) mood dysregulation.

Although etiologic questions remained regarding the patient’s perceptual disturbances, the new imaging findings showing signs of neurofibroma growth in his brain raised the CL team’s suspicion that the perceptual disturbances were from a medical source rather than early signs of a primary psychotic disorder. The prevalence of childhood psychosis and schizophrenia is 1/10,000, and given the patient’s negative family history, primary psychosis was lower on the differential [10]. Neurology was consulted for recommendations regarding immediate directed interventions for the NFI. Neurology recommended brain imaging. It was concluded that the patient continued to work with pediatric neurology outpatient for the management of NFI. With cognitive behavioral therapy, perceptual disturbances were likely to improve. EEG was recommended for outpatient.

The CL team aimed to address the patient’s hyperactivity and impulse control difficulties. The patient’s mother and the school staff shared collateral information regarding his poor concentration in the classroom being easily distracted when doing schoolwork and being hyperactive, which resulted in disruptive classroom behavior. The patient’s MRI findings of multiple non-enhancing foci in regions like the cerebellum and hippocampus may explain some of these behavioral concerns [11]. Current data suggest that the cerebellum can cause impairments in motor function within a temporal framework, resulting in patients being less able to inhibit behavior [12]. Our working diagnosis was ADHD, previously diagnosed. He was started on clonidine tablets 0.1 mg PO BID, his home dosage, to target reactivity symptoms and to help decrease sympathetic tone. He responded well with no side effects.

The CL team aimed to target the patient’s mood instability as he was preparing to be discharged from the hospital. The patient was struggling with his learning environment despite having a 504 plan, to ensure his ability to attend school despite his psychiatric disorders and behavioral outbursts. The team scheduled a discussion with his school and teachers about specific areas of weakness. As per his mother, the patient was in honors classes but performed poorly because he disliked attending school and often acted out. It was also important to address the patient’s bullying experiences in school, advise the school to counsel other students on their behavior, and advise our patient on how to respond to bullies and his low self-esteem that contributed to his repeated suicide attempts. It was recommended that he begin trauma-based psychotherapy to help build his self-confidence and reduce negative self-talk, thoughts of self-harm, and suicide attempts. He began this in the pediatric inpatient unit. In terms of family dynamics, the CL team provided psychoeducation to the patient’s mother about his new medications, therapy, and the need to increase support in his environment, as well as behavioral therapy to manage the "acting out." A family meeting was arranged as the optimal way to include all providers and support systems in confirming and implementing the care plan.

After eight days in the pediatric inpatient unit, the patient’s behavior improved and he denied SI/HI at the time, and the team/family concluded that it would be best for him to attend an intensive outpatient program (IOP), as he disliked the inpatient unit and was improving. His mother felt comfortable taking him to IOP and outpatient psychiatry/neurology. However, less than a month after discharge, the patient returned to the hospital for suicidal ideations with a plan and auditory perceptual disturbances, again requiring hospital admission. During this second visit, the CL team added methylphenidate 5 mg PO in the morning as an adjunct medication to clonidine 0.1 mg PO BID to help control his impulsive behaviors. His mother opted to start methylphenidate in a setting of medical observation. The patient was advised to follow up with outpatient psychiatry to begin an antidepressant to help mood for long-term follow-up and update of symptoms.

At that time, the CL team recommended that once the patient returned to the ambulatory care environment, his family would continue to work with the school to address bullying, as this was a consistent trigger for him. The patient was recommended a greater frequency of CBT, behavioral therapy, and outpatient neuropediatrics to evaluate the influence of NF1 on the patient's visual and auditory hallucinations. Since discharge, the patient has followed up with outpatient psychiatry and neurology, is currently using an antidepressant, attends group therapy, and has not returned to the hospital for suicidal ideations.

## Discussion

There is research regarding adult patients with neurofibromatosis but lacks information on children. In adult patients with neurofibromatosis, 46.5% were found to have at least one psychiatric comorbid diagnosis, most commonly mood disorders and anxiety disorders, as opposed to 20% of the general population [13]. NF1 is a complex and multisystem neurocutaneous disorder that can cause neuronal disturbances, leading to psychosis, which can be expressed through auditory and visual hallucinations [14].

While NFI is highly associated with psychiatric comorbidities, the etiology is not clear-cut. Several hypotheses include a high burden of disease, learning disabilities, and neurotransmitter dysregulation [15]. The activity of specific neurotransmitter pathways plays a role in NFI perceptual disturbances. In the 2021 study assessing psychiatric comorbidities and pharmacological treatment patterns in patients with NFI, it was observed that whole-brain serotonin levels were observed to be elevated in mice models of NF1 [15]. This is because neurofibromin, which is dysregulated in NF1, assists in the activation of serotonin receptor subtype six (5-HT6) [15]. With a decrease in the levels of neurofibromin, there is a lack of stimulation at the receptor resulting in decreased cyclic adenosine monophosphate (cAMP) and cAMP-responsive element-binding protein (CREB) levels, which are important in the regulation of cell survival, proliferation, and differentiation [16]. To treat the psychiatric symptoms of patients with NFI, antidepressants modulate the signaling pathways of monoamine neurotransmitters such as serotonin. The use of imipramine and fluoxetine has been shown to increase neurogenesis and improve behavioral symptoms in NF1 mice, likely due to the neural pathway affected [16]. These compounds directly stimulate the serotonin 5-HT receptor and therefore, correct dysregulated signaling to normalize the pathways.

There have been few case reports and research studies highlighting the association between psychiatric disorders in young patients with NFI. One study reported three childhood cases of patients with NFI and childhood psychosis, and the findings suggest that the simultaneous occurrence of NFI and psychosis may be more than coincidental [17]. A case report highlighted a triad in NFI of obsessive-compulsive disorder, ADHD, and Tourette’s syndrome [18]. A cohort study in Denmark reviewed data from 905 patients with NFI and 7614 controls matched on sex and age. The hazard ratio for the first psychiatric hospital contact was significantly higher in girls (4.19) and boys with NFI (5.02). Both sexes had an increased HR for developmental disorders, ADHD, intellectual disabilities, severe stress reactions, and psychosis [18]. In light of this finding, screening for early psychiatric disorders is imperative in the diagnosis and treatment of individuals with NFI [19]. This patient had hyperactivity and reactivity, similar to our pediatric patient who displayed behavioral disturbances and ADHD characteristics. However, this 22-year-old patient had also struggled with vocal tics since age 6 years, completing the triad.

It is therefore crucial to consider the psychiatric comorbidities that accompany NFI patients, especially those resulting from the interaction of genetics and environmental stressors [20]. Young children who have experienced trauma often have not yet developed the awareness, vocabulary, or cognitive processing to verbally communicate intense, complex emotions. As such, distress will often present behaviorally such as in the form of tantrums and aggression or regression [21]. As they move into adulthood, NF1 patients struggle often with anxiety, depression, stress, and low self-esteem, often due to a lack of coping skills and confidence fundamentally developed in childhood [21].

Neurofibromatosis type I (NFI) can cause a variety of medical problems and symptoms that can change over a person's life. In general, people with NFI have an average life expectancy, but in rare cases, severe tumors can shorten a person's lifespan. Some studies estimate that malignant cancers associated with NF1 can decrease life expectancy by 10 to 15 years [22]. For example, a 1995 study of 70 adult patients with NFI found that malignancy was the cause of death for more than half of the patients and that life expectancy decreased by about 15 years [22]. Therefore, it is important for patients to be informed about the increased risks that are associated with the disease to help screen early. Multidisciplinary teams are necessary including psychiatrists for perceptual disturbances, social workers for additional time in school, oncologists for increased cancer risk, etc. Furthermore, working with a pediatric neurologist is vital, especially a neurologist with a specialty in diseases like NFI. As our patient had ongoing pediatric neurologist specialty appointments, we recommended ongoing care with their neurologist for continuity. They will complete neuropsychological workups outpatient. In the future, we would recommend an inpatient workup.

The case presentation displays the complications in treating mood and visual/auditory hallucinations in patients with secondary causes of psychosis. Of note, it is vital to work with a multidisciplinary group to establish a clear etiology of the disease process. Further neuropsychiatric evaluation would have been important for our patient. While it was recommended outpatient, inpatient testing would provide guidance on management. Additionally, it poses a challenge when the patient's greatest triggers are at school, in a relatively uncontrolled environment. It is important to coordinate with the school barriers to bullying and prevent additional bullying episodes.

## Conclusions

This case report sheds light on how medical causes of perceptual disturbances can be overlooked and potentially treated incorrectly or incompletely before the root cause of a patient’s auditory or visual perceptual disturbances is identified. It is critical to include lesser-known medical causes of perceptual disturbances in the differential diagnosis. While neurofibromatosis I is a rare disorder, it is important to keep it in mind while determining etiologies of visual perceptual disturbances. Furthermore, involving a multidisciplinary team is vital for the quality of life of patients with NFI.
